# Use of CHADS_2_ and CHA_2_DS_2_-VASc Scores to Predict Subsequent Myocardial Infarction, Stroke, and Death in Patients with Acute Coronary Syndrome: Data from Taiwan Acute Coronary Syndrome Full Spectrum Registry

**DOI:** 10.1371/journal.pone.0111167

**Published:** 2014-10-24

**Authors:** Su-Kiat Chua, Huey-Ming Lo, Chiung-Zuan Chiu, Kou-Gi Shyu

**Affiliations:** 1 Graduate Institute of Clinical Medicine, College of Medicine, Taipei Medical University, Taipei, Taiwan; 2 Division of Cardiology, Department of Internal Medicine, Shin Kong Wu Ho-Su Memorial Hospital, Taipei, Taiwan; 3 Department of General Medicine, Shin Kong Wu Ho-Su Memorial Hospital, Taipei, Taiwan; 4 School of Medicine, Fu Jen Catholic University, New Taipei City, Taiwan; Azienda Ospedaliero-Universitaria Careggi, Italy

## Abstract

**Background:**

Acute coronary syndrome (ACS) patients have a wide spectrum of risks for subsequent cardiovascular events and death. However, there is no simple, convenience scoring system to identify risk of adverse outcomes. We investigated whether CHADS_2_ and CHA_2_DS_2_-VASc scores were useful tools to assess the risk for adverse events among ACS patients.

**Methods:**

This observational prospective study was conducted at 39 hospitals. Totally 3,183 patients with ACS were enrolled, and CHADS_2_ and CHA_2_DS_2_-VASc scores were calculated. The primary endpoint was occurrence of adverse event, including subsequent myocardial infarction, stroke, or death, within 1 year of discharge.

**Results:**

CHADS_2_ and CHA_2_DS_2_-VASc scores were significant predictors of adverse events in separate multivariate regression analyses. A Kaplan-Meier analysis of CHADS_2_ and CHA_2_DS_2_-VASc scores of ≥2 showed a higher rate of adverse events as compared with scores of <2 (*P*<0.001;log-rank test). CHA_2_DS_2_-VASc score was better than CHADS_2_ score in predicting subsequent adverse events; the area under the receiver operating characteristic curve increased from 0.66 to 0.70 (*p*<0.001). Patients with CHADS_2_ scores of 0 or 1 were further classified according to CHA_2_DS_2_-VASc score, using a cutoff value of 2. The rate of adverse events significantly differed between those with a score of <2 and those with a score of ≥2 (4.1% vs.10.7%, *P*<0.001).

**Conclusions:**

CHADS_2_ and CHA_2_DS_2_-VASc scores were useful predictors of subsequent adverse events in ACS patients.

## Introduction

Acute coronary syndrome (ACS) is diagnosed when patients present with unstable angina, non-ST-elevation myocardial infarction (MI), or ST-elevation MI. Such patients have a wide spectrum of risks for death and cardiovascular ischemic events.[1]–[3] Careful risk assessment of ACS patients helps clinicians determine prognosis and may therefore be useful in guiding management and providing valuable information to patients. [4], [5] To be clinically practical, a risk stratification model must be straightforward and use clinical risk factors that are readily ascertainable at hospital presentation.

Several scoring methods, including GRACE (Global Registry of Acute Coronary Events) [6], TIMI (Thrombolysis in Myocardial Infarction) [7], and PURSUIT (Platelet glycoprotein IIb/IIIa in Unstable angina: Receptor Suppression Using Integrillin Therapy) [8], are developed in order to distinguish ACS patients at the risk of adverse outcome, who may benefit most from aggressive therapies. However, there is no simple, convenience, and commonly accepted tool for assessing the risk of adverse clinical events such as MI, stroke, or death in patients with ACS. The CHADS_2_ (congestive heart failure; hypertension; age ≥75 years; type 2 diabetes; and previous stroke, transient ischemic accident [TIA], or thromboembolism [doubled]) score was originally used to estimate the risk of stroke in individuals with atrial fibrillation (AF) but is also a powerful predictor of stroke and death in patients with ischemic heart disease. [9], [10] A high score may be an independent marker of poor prognosis in cardiovascular disease.

The CHA_2_DS_2_-VASc score (congestive heart failure; hypertension; age ≥75 years [doubled]; type 2 diabetes; previous stroke, TIA, or thromboembolism [doubled]; vascular disease; age 65–75 years; and sex category) extends the CHADS_2_ score by considering additional risk factors for stroke and was recently recommended in a guideline for antithrombotic therapy in patients with AF or atrial flutter.[11]–[13] A previous study found that CHADS_2_ score could identify ACS patients at higher risk of adverse events and that the CHA_2_DS_2_-VASc and CHADS_2_ scores did not significantly differ in their power to predict mortality in ACS patients. [14] However, as compared with CHADS_2_ score, each additional component of the CHA_2_DS_2_-VASc score, such as peripheral vascular disease, female sex, and age 65–74 years, was associated with worse clinical outcomes in ACS patients. As compared with CHADS_2_ score, the CHA_2_DS_2_-VASc score is believed to have better prognostic predictive value for clinical outcomes. However, no published studies have investigated the association of CHADS_2_ and CHA_2_DS_2_-VASc scores with adverse event in patients with ACS. We compared the performance of CHADS_2_ and CHA_2_DS_2_-VASc scores in predicting subsequent MI, stroke, and death in patients with ACS.

## Methods

### Study design

In this prospective, nationwide, multicenter, non-interventional observational study, each participating site recruited 50–200 consecutive eligible patients. To ensure that the sample satisfactorily represented the ACS population, sites were selected by using data from the Scientific Committee of Taiwan Society of Cardiology. The accuracy of documentation was examined in 5% of case report forms at each recruiting site. Patient data collected included baseline characteristics such as risk factors for cardiovascular disease, clinical presentation, and in-hospital interventions, as well as medications prescribed and clinical outcomes. Participants were followed up at 3, 6, 9, and 12 months after discharge, and the data collected included medication use and clinical adverse events, including MI, stroke, and death.

### Patient recruitment

Patients were aged 20 years or older and were admitted to hospital within 24hours of presenting with symptoms of ACS. All patients who provided informed consent were eligible to be included in the study. Patients were excluded from this study if ACS was precipitated by comorbidity, such as trauma, if they were previously enrolled in this trial, or if they were participating in an investigational drug study.

This study was performed in accordance with the Declaration of Helsinki and good clinical practice. Ethics committee approval was obtained at all trial sites including China University Medical Hospital, Taoyuan General Hospital, Wan-Fang Hospital, Show Chwan Memorial Hospital, Chia-Yi Christian Hospital, Kuang Tien General Hospital, National Taiwan University Hospital, Cheng Ching Hospital, Sin Lau Hospital The Presbyterian Church of Taiwan, Tainan Municipal Hospital, Mackay Memorial Hospital, E-Da Hospital, Chi-Mei Hospital, Taichung Armed Forces General Hospital, Taipei Tzu Chi General Hospital, Kaohsiung Medical University Chung-Ho Memorial Hospital, Taichung Veterans General Hospital, Pingtung Christian Hospital, Lo-Tung Po-Ai Hospital, Far Eastern Memorial Hospital, National Cheng Kung University Hospital, National Taiwan University Hospital, Yun-lin Branch, Dalin Tzuchi General Hospital, Kee-lung Hospital, Taipei Veterans General Hospital, Cathay General Hospital, Kaohsiung Veterans General Hospital, Taipei Medical University Hospital, Shin Kong Wu Ho-Su Memorial Hospital, Changhua Christian Hospital, National Taiwan University Hospital, Chung Shan Medical University Hospita, Hualien Tzu Chi General Hospital, Mackay Memorial Hospital, Taitung Branch, Linkou Chang Gung Memorial Hospital, Hsin Chu General Hospital, Kaohsiung Chang Gung Memorial Hospital, Tri-Service General Hospital and Cheng-Hsin Hospital. Written informed consent was obtained from each patient.

### Definition of ACS

ACS was defined as a heterogeneous range of symptoms, from ST-elevation MI to unstable angina and non-ST-elevation MI, as previously described. [15] Briefly, ST-elevation MI was defined as presentation with acute chest pain, or overwhelming shortness of breath, together with persistent electrocardiographic ST elevation >1 mm in 2 or more contiguous leads, or with a new or presumed new left-bundle branch block pattern, on electrocardiography. Presentation with acute chest pain, or overwhelming shortness of breath, with no ST elevation but with classical rise and fall of at least one cardiac enzyme (troponin or MB fraction of creatine kinase) was defined as non-ST-elevation MI. Presentation with acute chest pain, or overwhelming shortness of breath, with neither ST elevation nor abnormal cardiac enzymes was defined as unstable angina.

### CHADS_2_, CHA_2_DS_2_-VASc and GRACE scores

CHADS_2_ score was calculated for all patients by assigning 1 point each for the criteria age ≥75 years, hypertension, diabetes mellitus, and heart failure and 2 points for the criterion previous stroke or TIA. For the CHA_2_DS_2_-VASc, 2 points were assigned for history of stroke/TIA or thromboembolism and age ≥75 years and 1 point each was assigned for the criteria age 65–75 years, history of hypertension, diabetes mellitus, heart failure, female sex, and vascular disease (defined as prior MI, complex aortic plaque, carotid disease, and peripheral artery disease, including intermittent claudication, previous surgery or percutaneous intervention for the abdominal aorta or vessels of the lower extremities, and arterial and venous thrombosis). [11], [12] The cutoff values used for grouping CHADS_2_ and CHA_2_DS_2_-VASc scores were determined according to values used in earlier studies of the risk of stroke and atrial properties. [12], [16], [17] Besides, the GRACE risk score [6] (age, Killip class, heart rate, systolic BP, ST-segment deviation and cardiac arrest at admission, elevated biomarkers of myocardial necrosis, and baseline creatinine level) were also calculated from data collected at admission.

### Statistical analyses

Sample size for the Taiwan ACS full-spectrum registry was calculated as follows. There are about 50,000 new ACS cases per year in Taiwan. On the basis of the known background incidence rate of 0.0025, a sample of 2,395 patients would achieve 80% power to detect an additional incidence rate of 0.003, with a precision of 0.2% and a 95% confidence interval (CI). Assuming a dropout rate of 20%, a sample of 3,000 was considered adequately representative.

Parameters were summarized using mean, median, standard deviation, and interquartile range, where appropriate, for continuous data, and counts or percentages for categorical data. For comparability between groups, the chi-squared test was used for categorical variables, and analysis of variance (ANOVA) was used for continuous variables. Univariate associations of variables with adverse events, including subsequent MI, stroke, and death, were assessed with multivariate logistic regression. For each variable, the hazard ratio (HR), 95% CI, and *P* value are provided. The cumulative adverse events curves were constructed according to the Kaplan-Meier method. All statistical tests were two-sided, and a p value of <0.05 was considered to indicate statistical significance. Analyses were done using a time to first event approach, without double counting of events in analyses involving composite endpoints. Patients lost to follow-up were censored at the time of last contact, and their vital status was classified as alive and event-free at that time. We assessed the predictive accuracy of CHADS_2_ and CHA_2_DS_2_-VASC scores by using the receiver operating characteristic (ROC) curve analysis. The areas under the ROC (AUCs) for these 2 indices were compared by using De Long’s method. Statistical analysis was performed using SAS software version 9.2 (SAS Institute Inc., Cary, NC, USA).

## Results

### Clinical characteristics of participants and predictors of acute coronary syndrome

During the period from October 2008 through January 2010, 3,183 eligible patients were enrolled at 39 hospitals in Taiwan. The study population had a mean age of 64 years (range, 20–101 years) and comprised 2,483 (78%) men and 700 (22%) women. Of these 3,183 patients, 2,016 (63%) had hypertension, 1,138 (36%) had diabetes mellitus, 1,235 (39%) had hyperlipidemia, 172 (5%) had a history of congestive heart failure, and 287 (9%) had a history of stroke or TIA. In addition, 367 (12%) patients had vascular disease, including 315 with a history of MI and 71 with peripheral vascular disease.

Table 1 shows the baseline clinical characteristics of the patients, stratified using a cutoff value of 2 on the CHADS_2_ and CHA_2_DS_2_-VASC indices. The burden of previous cardiovascular disease was somewhat greater in patients with a CHADS_2_ or CHA_2_DS_2_-VASC score of ≥2. Hypertension was the most important risk factor among patients with a CHADS_2_ or CHA_2_DS_2_-VASC score of ≥2. CHADS_2_ and CHA_2_DS_2_-VASC scores were inversely associated with ST-elevation MI; however, non-ST-elevation MI and unstable angina were more frequent among those with a CHADS_2_ or CHA_2_DS_2_-VASC score of ≥2. CHADS_2_ and CHA_2_DS_2_-VASC scores were inversely associated with primary percutaneous coronary intervention and the level of the MB fraction of creatine kinase. Participants with higher CHADS_2_ and CHA_2_DS_2_-VASC scores were more likely to present with a high Killip class and greater LV systolic dysfunction. There was no significant difference in drug regimen at discharge (including use of dual antiplatelet therapy, angiotensin-converting enzyme inhibitors or angiotensin receptor blockers, β-blockers, and statins) between patients with a CHADS_2_ or CHA_2_DS_2_-VASC score of <2 and those with higher scores.

**Table 1:** Baseline characteristics of patients stratified using a cutoff value of 2 for CHADS_2_ and CHA_2_DS_2_-VASc scores.

Variable	CHADS_2_ score			CHA_2_DS_2_-VASc score		
	<2 (n = 1,805)	≥2 (n = 1,378)	*P* value	<2 (n = 1,242)	≥2 (n = 1,941)	*P* value
Age, years	58.3±12.1	71.5±12.1	<0.001	53.4±9.4	70.9±11.6	<0.001
Age 65–75 years	439 (24.3)	313 (22.7)	0.16	122 (9.8)	630 (32.5)	<0.001
Age ≥75 years	129 (7.1)	679 (49.3)	<0.001	0 (0)	808 (41.6)	<0.001
Male	1,546 (85.7)	937 (68.0)	<0.001	1,196 (96.3)	1,287 (66.3)	<0.001
Medical History						
Current smoker	946 (52.4)	367 (26.6)	<0.001	774 (62.8)	539 (27.6)	<0.001
Hypertension	734 (41.2)	1,282 (93.5)	<0.001	421 (33.9)	1,595 (82.2)	<0.001
Diabetes	208 (11.6)	930 (67.7)	<0.001	122 (9.8)	1016 (52.3)	<0.001
Hyperlipidemia	599 (33.6)	636 (46.4)	<0.001	402 (32.8)	833 (42.9)	<0.001
Congestive heart failure	12 (0.7)	160 (11.6)	<0.001	3 (0.2)	169 (8.7)	<0.001
Previous CAD	275 (15.2)	507 (36.8)	<0.001	115 (9.3)	667 (34.3)	<0.001
Previous myocardial infarction	107 (21.3)	208 (31.9)	<0.001	25 (2.0)	290 (14.9)	<0.001
Previous stroke/TIA	0 (0)	287 (20.8)	<0.001	0 (0)	287 (14.8)	<0.001
Peripheral arterial disease	13 (0.7)	58 (4.2)	<0.001	2 (0.2)	69 (3.6)	<0.001
Vascular disease§	119 (6.6)	259 (18.8)	<0.001	27 (2.2)	340 (17.5)	<0.001
History of atrial fibrillation	30 (1.7)	73 (5.3)	<0.001	8 (0.6)	95 (4.9)	<0.001
Chronic kidney disease	314 (17.4)	609 (44.2)	<0.001	171 (13.8)	752 (38.7)	<0.001
COPD	40 (2.2)	83 (6.0)	<0.001	14 (1.1)	109 (5.6)	<0.001
Clinical presentation						
ST-elevation MI	1,120 (62.0)	583 (42.3)	<0.001	822 (66.2)	881 (45.4)	<0.001
Non-ST elevation MI	361 (20.0)	489 (35.5)	<0.001	222 (17.9)	628 (32.4)	<0.001
Unstable angina	324 (18.0)	306 (22.2)	<0.001	198 (15.9)	432 (22.3)	<0.001
Killip class ≥III at admission	238 (13.2)	289 (21.0)	<0.001	135 (10.9)	392 (20.2)	<0.001
CK-MB maximum, median ug/L	76.8±131.3	46.6±81.3	<0.001	83.2±138.2	51.5±92.5	<0.001
LVSD (LVEF<40%)	171 (9.5)	220 (16.0)	<0.001	102 (8.2)	289 (14.9)	<0.001
Procedures						
Fibrinolysis therapy	33 (2.5)	22 (2.6)	0.89	22 (2.3)	33 (2.7)	0.68
PCI	1,588 (88.1)	1,092 (79.5)	<0.001	1,111 (89.6)	1,569 (81.0)	<0.001
Primary PCI	1,016 (56.3)	490 (35.6)	<0.001	725 (58.4)	781 (40.2)	<0.001
Rescue PCI	29 (2.1)	12 (1.4)	0.26	21 (2.2)	20 (1.6)	0.34
CABG	49 (2.7)	57 (4.1)	0.03	26 (2.1)	80 (4.1)	0.002
Medication at discharge						
Dual antiplatelet therapy	1,351 (74.8)	1,034 (75.0)	0.93	923 (74.3)	1,462 (75.3)	0.53
ACEi/ARB	1,157 (64.1)	848 (61.5)	0.07	769 (61.9)	1,236 (63.9)	0.32
β-blockers	988 (54.7)	712 (51.7)	0.05	653 (52.6)	1,047 (53.9)	0.45
Statin therapy	1,091 (60.4)	833 (60.4)	1.00	745 (60.0)	1,179 (60.7)	0.67
VKA	34 (1.9)	33 (2.4)	0.89	26 (2.1)	41 (2.1)	0.69

Values are presented as number (%) or mean ± SD.

ACEi, angiotensin-converting enzyme inhibitors; ARB, angiotensin receptor blockers; CABG, coronary artery bypass grafting; CAD, coronary artery disease; CK-MB, MB fraction of creatine kinase; COPD, chronic obstructive pulmonary disease; LVEF, left ventricular ejection fraction; LVSD, left ventricle systolic dysfunction; MI, myocardial infarction; PCI, percutaneous coronary intervention; TIA, transient ischemic attack; VKA, vitamin K antagonist.

§ Vascular disease defined as previous myocardial infarction or peripheral arterial obstructive disease.

### CHADS_2_ and CHA_2_DS_2_-VASc scores and prediction of subsequent MI, stroke, and death

**Figure 1:**
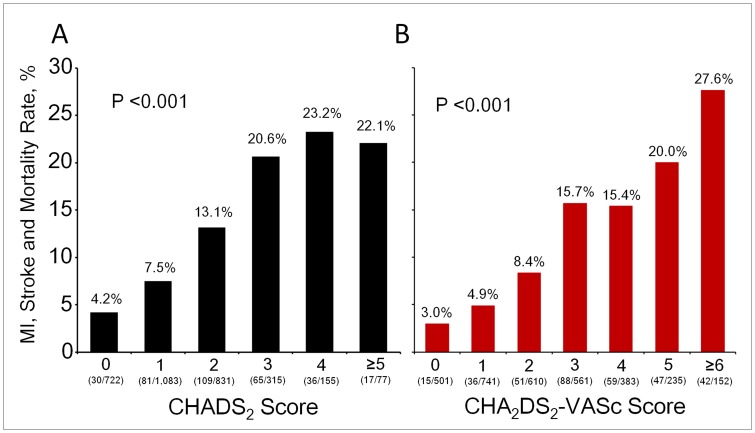
Rates of adverse events, including myocardial infarction (MI), stroke, or death, according to CHADS_2_ and CHA_2_DS_2_-VASc scores. The rate of MI, stroke, or death increased as CHADS_2_ (A) and CHA_2_DS_2_-VASc (B) scores increased.

**Figure 2:**
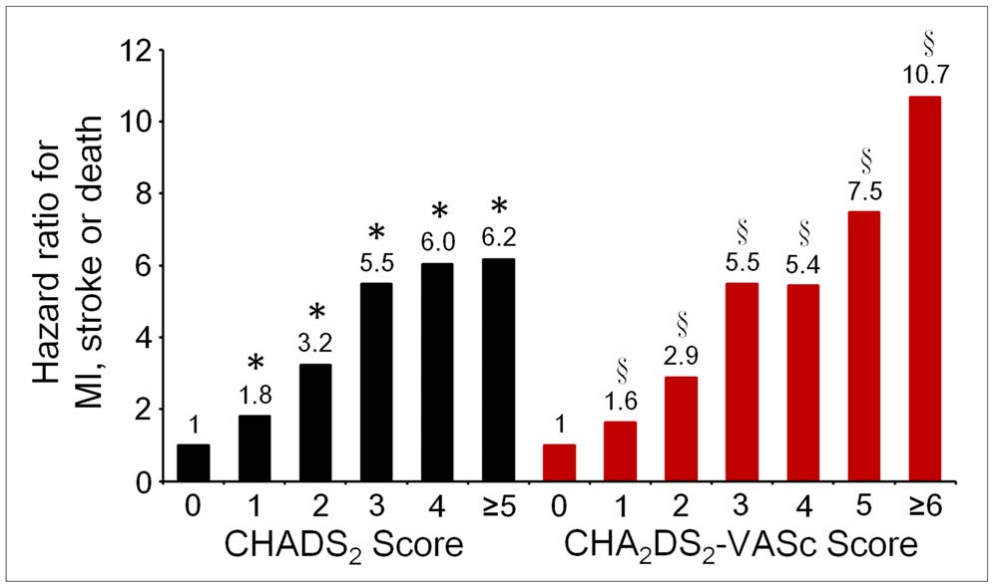
Adjusted hazard ratios for the composite endpoint myocardial infarction (MI), stroke, or death, in relation to CHADS_2_ or CHA_2_DS_2_-VASc scores, in patients with acute coronary syndrome. The risk of MI, stroke, or death progressively increased with each unit increase in CHADS_2_ and CHA_2_DS_2_-VASc scores. The reference groups are patients with scores of 0. * And § are defined as p<0.001 vs. CHADS_2_ and CHA_2_DS_2_-VASc scores of 0, respectively.

Rates of MI, stroke, and death increased with increasing CHADS_2_ and CHA_2_DS_2_-VASc scores (Fig. 1). Figure 2 shows the HRs for adverse events in relation to CHADS_2_ and CHA_2_DS_2_-VASc scores in patients with ACS. The risk of adverse events progressively increased as CHADS_2_ and CHA_2_DS_2_-VASc scores increased. Clinical outcomes during follow-up, in relation to CHADS_2_ and CHA_2_DS_2_-VASC scores at the cutoff value of 2, are summarized in Table 2. Patients with CHADS_2_ or CHA_2_DS_2_-VASC scores of >2 had higher risks of stroke and death. Overall, a CHADS_2_ or CHA_2_DS_2_-VASC score of >2 was associated with higher risks of MI, stroke, and death during follow-up.

**Table 2:** Clinical outcomes during follow-up stratified using a cutoff value of 2 for CHADS_2_ and CHA_2_DS_2_-VASc scores.

Variable	CHADS_2_ score			CHA_2_DS_2_-VASc score		
	<2 (n = 1,805)	≥2 (n = 1,378)	*P* value	<2 (n = 1,242)	≥2 (n = 1,941)	*P* value
Myocardial infarction or stroke	61 (3.4)	89 (6.5)	<0.001	40 (3.2)	110 (5.7)	0.001
Myocardial infarction	49 (2.7)	52 (3.8)	0.09	32 (2.6)	69 (3.6)	0.13
Stroke	12 (0.7)	39 (2.8)	<0.001	8 (0.6)	43 (2.2)	<0.001
Death	52 (2.9)	159 (11.5)	<0.001	12 (1.0)	199 (10.3)	<0.001
Myocardial infarction, stroke, or death	111 (6.2)	227 (16.5)	<0.001	51 (4.1)	287 (14.8)	<0.001

Values are presented as number (%) or mean ± SD.

At a cutoff value of 2, a higher CHADS_2_ or CHA_2_DS_2_-VASC score was significantly associated with rates of MI, stroke or death, before and after adjustment for potential confounders (Table 3). The risk of subsequent MI, stroke, or death increased with every unit increase in CHADS_2_ or CHA_2_DS_2_-VASC score. After adjustment, the HR for future MI, stroke, or death per unit increase in CHADS_2_ and CHA_2_DS_2_-VASC scores was 1.44 (95% CI 1.30–1.58, p<0.001) and 1.36 (95% CI 1.26–1.46, p<0.001), respectively.

**Table 3:** Hazard ratios for myocardial infarction, stroke, or death according to baseline CHADS_2_ and CHA_2_DS_2_-VASc scores.

Characteristic	Unadjusted, HR (95% CI)	P Value	Adjusted, HR (95% CI)§	P Value
Myocardial infarction or stroke				
CHADS_2_≥2 vs <2	2.03 (1.46–2.81)	<0.001	1.87 (1.28–2.72)	0.001
CHADS_2_ score†	1.28 (1.13–1.44)	<0.001	1.25 (1.08–1.44)	0.002
CHA_2_DS_2_-VASc ≥2 vs <2	1.72 (1.19–2.48)	0.004	1.63 (1.10–2.47)	0.02
CHA_2_DS_2_-VASc score†	1.18 (1.08–1.29)	<0.001	1.18 (1.06–1.31)	0.002
Death				
CHADS_2_≥2 vs <2	4.40 (3.12–6.06)	<0.001	3.17 (2.24–4.47)	<0.001
CHADS_2_ score†	1.74 (1.58–1.92)	<0.001	1.60 (1.41–1.80)	<0.001
CHA_2_DS_2_-VASc ≥2 vs <2	11.5 (6.42–20.8)	<0.001	8.52 (4.48–16.2)	<0.001
CHA_2_DS_2_-VASc score†	1.62 (1.50–1.75)	<0.001	1.55 (1.42–1.70)	<0.001
Myocardial infarction, stroke or death				
CHADS_2_≥2 vs <2	2.74 (2.19–3.41)	<0.001	2.33 (1.81–2.99)	<0.001
CHADS_2_ score†	1.52 (1.40–1.65)	<0.001	1.44 (1.30–1.58)	<0.001
CHA_2_DS_2_-VASc ≥2 vs <2	3.42 (2.58–4.53)	<0.001	2.98 (2.17–4.07)	<0.001
CHA_2_DS_2_-VASc score†	1.41 (1.33–1.50)	<0.001	1.36 (1.26–1.46)	<0.001

HR = hazard ratio; other abbreviations as in Tables 1 and 2.

§ Adjusted for all clinical variables in Table 1 (except the 5 or 7 variables included in the CHADS_2_ and CHA_2_DS_2_–VASc scoring systems, respectively), LVEF, Killip class, chronic kidney disease and medication at discharge.

† Per unit increase in the original 6- or 8-criteria CHADS_2_ and CHA_2_DS_2_-VASc scoring systems, respectively.

**Figure 3:**
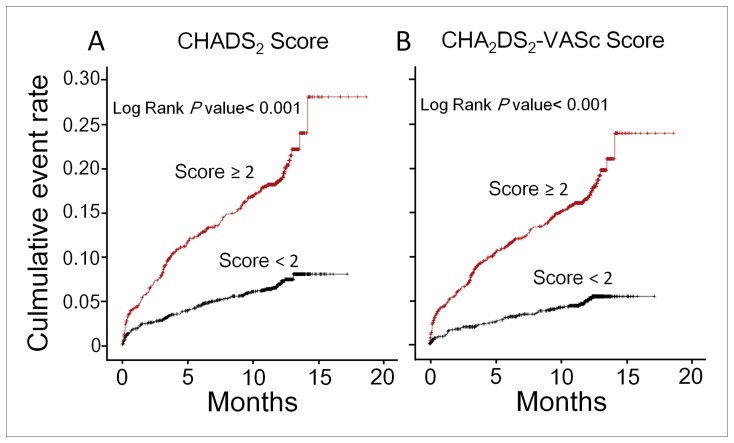
Kaplan-Meier curves for the time to the composite endpoint of myocardial infarction (MI), stroke, or death, according to CHADS_2_ and CHA_2_DS_2_-VASc scores. Survival analysis showed that a CHADS_2_ score of ≥2 was associated with a higher event rate than a score of <2 (*p*<0.001; log-rank test) (A). In addition, a CHA_2_DS_2_-VASc score of ≥2 was a significant predictor of adverse events (*p*<0.001; log-rank test) (B).

**Figure 4:**
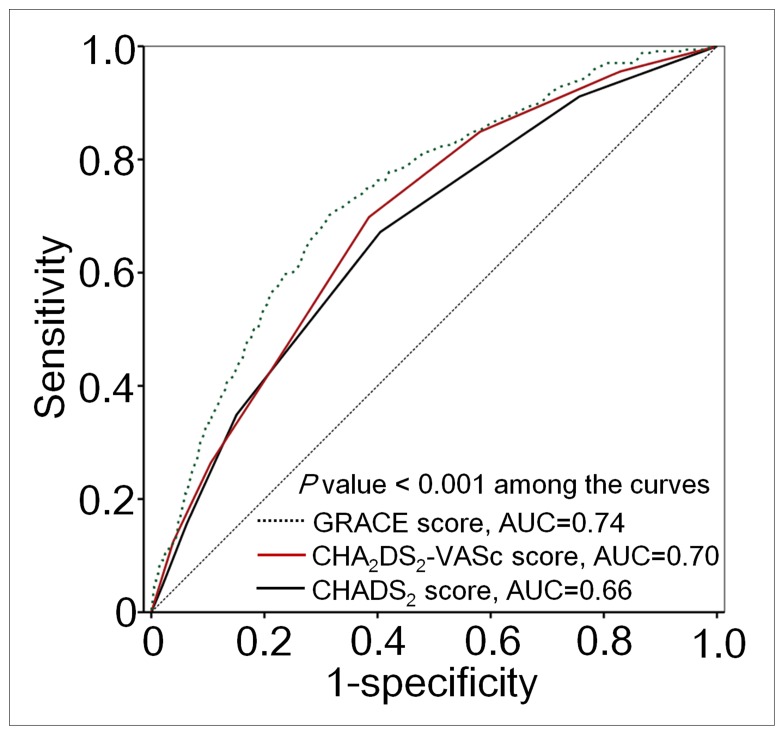
Receiver operating characteristic (ROC) curves for CHADS_2_, CHA_2_DS_2_-VASc and GRACE scores predicting myocardial infarction (MI), stroke, or death. Diagnostic performance in predicting MI, stroke, or death was better for CHA_2_DS_2_-VASc score than for CHADS_2_ score. The area under the ROC curve (AUC) increased from 0.66 to 0.70, and the difference was statistically significant (*p*<0.001). Besides, the diagnostic accuracy in predicting adverse events was better for GRACE score than for CHA_2_DS_2_-VASc score (AUC 0.74 vs. 0.70, *p*<0.001).

Kaplan-Meier survival analysis revealed that patients with a CHADS_2_ score of ≥2 had a higher rate of MI, stroke, or death than did those with lower CHADS_2_ scores (*P*<0.001, log-rank test; Fig. 3A). Furthermore, a CHA_2_DS_2_-VASc score of ≥2 was also a significant predictor of an adverse event (*P*<0.001, log-rank test; Fig. 3B). However, CHA_2_DS_2_-VASc score had better diagnostic performance in predicting the composite endpoint subsequent MI, stroke, or death, as compared with CHADS_2_ score. The AUC increased from 0.66 to 0.70, and the difference was statistically significant (p<0.001), as shown in Figure 4. The GRACE risk score (AUC = 0.74) had better diagnostic accuracy in predicting adverse events compared with CHA_2_DS_2_-VASc score. (p<0.001).

### CHA_2_DS_2_-VASc score and subsequent adverse events in patients with a CHADS_2_ score of 0 or 1

**Figure 5:**
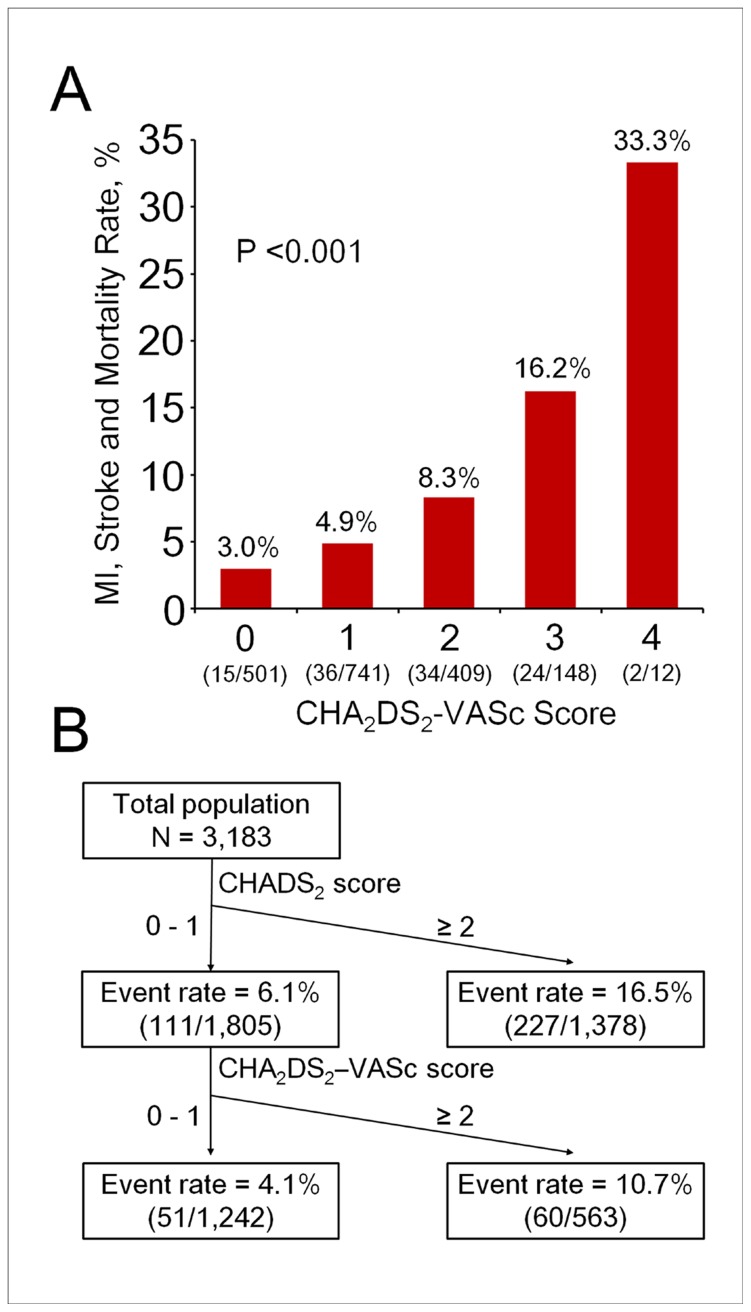
Flowchart of adverse event rates and risk scores in the patients with CHADS_2_ score of 0 or 1. (A) Rate of MI, stroke, or death in patients with a CHADS_2_ score of 0 or 1, according to CHA_2_DS_2_-VASc score. The rate of myocardial infarction (MI), stroke, or death progressively increased, from 3.0% to 33.3%, with increasing CHA_2_DS_2_-VASc score. (B) The flowchart shows the rate of MI, stroke, or death in patients stratified by CHADS_2_ and CHA_2_DS_2_-VASc scores.

A subgroup analysis of the 1805 patients with CHADS_2_ scores of 0 or 1 revealed that 111 (6%) had a subsequent MI, stroke, or death, and the rate progressively increased from 3.0% (in patients with CHA_2_DS_2_-VASc scores of 0) to 33.3% (in patients with CHA_2_DS_2_-VASc scores of 4) (p<0.001; Fig. 5A). Using a CHA_2_DS_2_-VASc score of 2 as the cutoff point, patients with a score of ≥2 had a higher event rate than did those with a CHA_2_DS_2_-VASc score of <2 (10.7% vs. 4.1%, p<0.001; Fig. 5B).

## Discussion

### Principal findings

In this study of a cohort of patients with ACS, CHADS_2_ and CHA_2_DS_2_-VASc scores were helpful and convenient indices for predicting subsequent MI, stroke, or death. CHAD_2_DS_2_-VASc score was useful for further risk stratification for clinical outcome among patients with CHADS_2_ scores of 0 or 1. The usefulness of this simple and popular scoring system allows clinicians to summarize the overall risk of MI, stroke, and death in patients with ACS.

### CHADS_2_ score in patients with ACS

The CHADS_2_ score is a risk index for predicting stroke in patients with AF and can be used to guide anticoagulation therapy. [18], [19] A previous study found that CHADS_2_ score predicted clinical outcomes in ACS patients with and without AF. [14] It is reasonable to assume that CHADS_2_ score is valuable in ACS, since each of its components is a prognostic risk factor for ischemic heart disease [20], [21] and stroke. [22], [23] Furthermore, a previous study reported that heart failure, hypertension history, increasing age, and diabetes were independent risk factors for long-term mortality in patients with acute MI. [20] This agrees with our finding that 7% of the present ACS patients with a CHADS_2_ score of <2, vs. 17% with a score of ≥2, had subsequent MI, stroke, or death. Using multivariate models, we found that CHADS_2_ score was a powerful predictor of subsequent adverse events after ACS. These findings extend the usefulness of the CHADS_2_ score in predicting clinical outcomes in patients with ACS. The CHADS_2_ score may help identify treatable underlying conditions in patients with ACS, thereby decreasing subsequent risk.

### CHA_2_DS_2_-VASc scores in patients with ACS

The new CHA_2_DS_2_-VASc score extends the CHADS_2_ score by adding the criteria age 65–74 years, vascular disease, and female sex, which increases the predictive value of the CHADS_2_ for thromboembolic events with low event rates in low-risk patients. [11], [12] A previous study found that CHADS_2_ and CHA_2_D_2_-VASc scores did not significantly differ in relation to prediction of mortality in ACS patients; however, it is important to note that these scoring systems were developed to predict stroke and thromboembolism, not mortality. [14] We found that, as compared with CHADS_2_ score, CHA_2_DS_2_-VASc score had better diagnostic performance in predicting subsequent adverse events. In addition, the AUC significantly increased, from 0.66 to 0.70. Moreover, CHA_2_DS_2_-VASc score could further predict risk of subsequent MI, stroke, or death in ACS patients with CHADS_2_ scores of 0 and 1.

The impact of female sex on ACS has been investigated: as compared with men, women had more complications during hospitalization and a higher mortality rate.[24]–[26] Women and men with ACS had a different clinical outcome, which reflects pathophysiologic and anatomic differences between sexes. [26] Peripheral vascular disease is often complicated by ischemic episodes, not only in peripheral circulation but also in coronary and cerebral vessels. [27], [28] The rate of cardiovascular mortality among patients with peripheral vascular disease was three-fold that of age-matched controls. [29], [30] Furthermore, the presence of peripheral vascular disease in conjunction with ACS is associated with substantial mortality and morbidity. [31] Given that age does not have a binary effect on the risk of adverse events and that age ≥75 years was associated with high risk, it is understandable that the criterion age 65–74 years, in combination with another risk factor, was associated with increased risk in ACS patients.^23^ It was estimated that 60% of ACS cases were people aged ≥65 years and that 30% were people aged ≥75 years. In addition, as many as 80% of deaths related to ACS occur in patients aged ≥65 years. [32], [33] Taken together, these findings suggest that the risk of subsequent MI, stroke, or death increases with the combination of these additional risk factors in the CHA_2_DS_2_-VASc score.

The more complicated GRACE score provided a better prediction for subsequent adverse events than the simpler CHA_2_DS_2_-VASc score according to the ROC curve analysis. However, one great advantage of the CHA_2_DS_2_-VASc score is that it provides a comprehensive, convenience, and fast method for clinical physician in risk evaluation. No calculators or computers are needed for the risk stratification.

### Clinical implications

The CHADS_2_ scoring system was a simple tool for predicting adverse events among ACS patients. A CHADS_2_ score of ≥2 was associated with a 16.5% risk of adverse events in ACS patients. Moreover, the more detailed CHA_2_DS_2_-VASc scoring system could further discriminate the risk of developing adverse events among patients with a CHADS_2_ score of 0 or 1. The clinical utility of the CHA_2_DS_2_-VASc score should be emphasized, as it was generally believed that patients with a CHADS_2_ score of 0 or 1 were at low risk; however, among this subgroup, those with a CHA_2_DS_2_-VASc score of 4 have a rate of adverse events as high as 33.3%. These findings suggest that CHA_2_DS_2_-VASc score is useful in identifying ostensibly low-risk patients who are at risk of adverse events and optimizing management of such patients so as to lower such risk. However, this requires confirmation in a large-scale prospective trial.

### Study limitations

This study had several limitations. Patients at other sites might have risk profiles and subsequent outcomes that vary depending on differences in ACS treatment. In addition, adverse events among the present participants would have been missed if such episodes occurred at other hospitals. The incidences of adverse events in the present study may have been underestimated, which would have biased the results against a significant association of CHADS_2_ and CHA_2_DS_2_-VASc scores with adverse events in the present study.

## Conclusion

CHADS_2_ and CHA_2_DS_2_-VASc scores can be used to estimate the risk of clinical adverse events in patients with ACS. Among patients with CHADS_2_ scores of 0 or 1, the CHA_2_DS_2_-VASc score was helpful in identifying patients who were at higher risk. These scoring systems could lead to optimization of therapy, which might reduce risks of subsequent adverse events.
